# Prevalence and awareness of obesity and related husbandry practices in Estonian rabbits, guinea pigs, and rats

**DOI:** 10.1017/awf.2025.10042

**Published:** 2025-10-10

**Authors:** Mariin Pantelejev, Kristin Tõnise

**Affiliations:** Estonian University of Life Sciences, FR Kreutzwaldi 1, Tartu 51006, Estonia

**Keywords:** Animal welfare, body condition score, feeding, individual housing, nutrition, rodent, weight gain

## Abstract

Obesity has significant implications regarding the welfare of companion animals. Data regarding obesity in exotic companion mammals (ECM) are sparse. The aim of this study was to investigate obesity in pet rabbits (*Oryctolagus cuniculus*), guinea pigs (*Cavia porcellus*), and rats (*Rattus norvegicus*) in Estonia, and to survey husbandry practices and owner awareness. Husbandry data were collected from patients visiting the Estonian University of Life Sciences’ small animal clinic via anonymous questionnaires over an eleven-month period. Three hundred and fifty-one questionnaire responses and body condition score (BCS) data for 177 patients (71 rabbits, 73 guinea pigs, 33 rats) were collected. Twenty-eight percent of rabbits, 23% of guinea pigs and 28% of rats were overweight (BCS > 3/5). Male rats were more likely to be overweight than females and there was a negative correlation between age and body condition. There was an increased likelihood of male guinea pigs being underweight. Owner questionnaires revealed that 20% of rabbit owners, 14% of guinea pig owners and 11% of rat owners believed their pets to be overweight while 58% of owners had not received husbandry advice from a veterinarian. Obesity is a significant welfare issue in the Estonian ECM population and several detrimental husbandry practices were identified, including inappropriate feeding, insufficient physical activity, individual housing. Further studies might investigate veterinarian awareness of the issues at hand and tendencies for other species.

## Introduction

Excess weight is generally considered to be the most common nutritional disorder in companion animals. There has been a paucity of surveys related to exotic companion mammals (ECMs), but among dogs (*Canis familiaris*) and cats (*Felis catus*), 24–44% of the adult population is estimated to be overweight (Michel & Bonnet 2012). In a first opinion practice-based survey in the UK, almost 75% of rabbits (*Oryctolagus cuniculus*) were assessed as being in ideal body condition by vets with 7.6% being overweight (Courcier *et al.* 2012). A study of a veterinary practice in Edinburgh, UK, found 12% of rabbit patients to be overweight, whereas another UK study found the prevalence was 35% (Sweet *et al.* 2013; Thompson *et al.* 2019). In a 2019 study of commonly diagnosed disorders in guinea pigs (*Cavia porcellus*) in the UK, obesity was diagnosed in just 2.14% of cases (O’Neill *et al.* 2024). Obesity has also been named the most common nutritional disease in pet rats (*Rattus norvegicus*) (Frohlich 2020).

Excessive bodyweight presents a significant welfare issue for ECMs. For example, rabbits with experimentally induced obesity show myocardial hypertrophy, higher resting heart rates, and metabolic syndrome, which is characterised by hypertension, hyperinsulinaemia, hyperglycaemia and hypertriglyceridaemia (Carroll *et al.* 1996). Similar effects are seen in rats, mice (*Mus musculus*), and guinea pigs (Wong *et al.* 2016). In rabbits, obesity may impede the consumption of caecotrophs if the animal is unable to reach the anus with failure to consume caecotrophs associated with potential amino acid and vitamin deficiency (Campbell-Ward 2012). Obesity may also predispose rabbits to dystocia and result in difficulties grooming with the creation of skin folds that trap in moisture and bacteria, resulting in dermatitis, myasis and urine scalding (Hess & Tater 2012). Pododermatitis, pregnancy toxaemia, hepatic lipidosis and gastrointestinal stasis are further associations with excess bodyweight in rabbits (Meredith 2012).

Excess body fat in guinea pigs is known to cause subconjunctival lipid deposition or ‘fatty eye’ (Minarikova *et al.* 2015) as well as having been found to negatively affect fertility in females, reducing pregnancy rates and litter sizes (Michel & Bonnet 2012). Urolithiasis is a common health problem in guinea pigs and has been linked with obesity and sedentarism in other small mammal species, such as rabbits (Clauss & Hatt 2017; Edell *et al.* 2022). Obesity has also been identified as a possible risk factor for osteoarthritis in guinea pigs (Keeble 2021).

In rats, preventing weight gain after ovariectomy has been found to significantly decrease the incidence of mammary gland tumours (Wellberg *et al.* 2022) while bodyweight has also been shown to correlate positively with the incidence of pituitary tumours in rats of both sexes (Gries & Young 1982), not to mention with islet cell and lipomatous tumours in males and fibromatous tumours in females (Turnbull *et al.* 1985). Higher calorie intake as well as higher fat intake have been found to increase the incidence of mammary tumours in both rats and mice (Freedman *et al.* 1990) and higher bodyweight has been associated with increased mortality in rats (Turnbull *et al.* 1985).

Excessive bodyweight may be an indication of poor husbandry and nutrition. There may be a lack of awareness regarding dietary and enrichment or activity requirements among ECM owners while veterinarians may not feel confident enough to make recommendations. Previous surveys in other countries have revealed shortcomings in owners’ knowledge of husbandry requirements in rabbits (Rooney *et al.* 2014; Mayer *et al.* 2017; McMahon & Wigham 2020), guinea pigs (Harrup & Rooney 2020; Wills 2020) and rats (Neville *et al.* 2021).

## Materials and methods

### Selection of sampled animals and data collection

Veterinarians working at the Estonian University of Life Sciences small animal clinic were instructed to determine the body condition score (BCS) for all rabbits, guinea pigs, rats, and mice that were brought to the clinic between May 1, 2022, and April 1, 2023, in addition to the data that would normally be collected for each patient (age, sex, neuter status, weight, physical examination, complaints). To determine BCS, UK Pet Food’s ‘Pet Size-O-Meter’ charts were used for rabbits (UK Pet Food 2024b) and guinea pigs (UK Pet Food 2024a). For rats, the scoring was based on Hickman and Swan’s (2010) method. BCS systems use a five-point integer scale, however, for the purposes of this study, half-integer scores were also included, since the majority of assessing clinicians routinely made use of them. Veterinarians were provided with illustrations and instructions for using these systems.

These data were recorded either on specifically designed forms (see Appendix 1; Supplementary material) or via the clinic’s online patient database, Provet Cloud, with the form then filled retrospectively, based on the digital patient record.

### Selection of owners and questionnaire

A nine-question, multiple-choice, anonymous questionnaire in either Estonian or English was distributed for voluntary completion by rabbit, guinea pig, rat, and mouse owners visiting the clinic (see Appendix 1; Supplementary material). This included questions regarding owners’ assessment of their pets’ body condition, whether they had received a body condition assessment or husbandry advice from a veterinarian, feeding practices and husbandry, e.g. whether pets were group-housed and whether they were allowed to roam or were kept in a cage. The questions were constructed in such a way as to enable respondents keeping multiple individuals of the same same species to select multiple answers. The questionnaire was provided on paper for voluntary completion prior to the clinic visit as well as being made available online on Google Forms and posted on several Estonian rodent and rabbit Facebook groups. Participants were instructed to fill out the questionnaire once per animal species, provided that they were currently the owner of a rabbit, guinea pig, rat and/or mouse and were living in Estonia. Questionnaires were made available from May 1, 2022, to December 31, 2022 and were collected separately from patient data, i.e. not matched to patients.

### Data handling and statistical analysis

Data were collected and stored online in Google Forms, on paper, or in the electronic patient database ProVet Cloud. Mice were excluded from data analysis due to an insufficient sample size (one mouse owner and none among the patients). For analysis, all data were entered into Microsoft® 365 Excel, where choice and qualitative responses were coded. As data were not normally distributed, non-parametric statistic tests (Kruskal-Wallis and Mann-Whitney *U*) were used to assess differences in activity levels between individually and group-housed animals. Associations between age, sex, spay/neuter status and being overweight were evaluated with the Fisher’s exact test. Age and BCS were also compared with the Spearman correlation test. Results were assessed for significance defined as *P* < 0.05. Odds ratios (OR) and 95% confidence intervals (CI) were calculated. Statistical analyses were performed using Microsoft® Excel, R 4.3.0 (R Core Team 2023), and OpenEpi (Dean *et al.* 2013). Figures were constructed using Microsoft® Excel.

### Ethical considerations

In accordance with institutional and national guidelines, non-invasive patient surveys and anonymous questionnaires did not require ethical review. Body condition observations are a part of regular, non-invasive clinical examinations for patients. Consent for using the patients’ data was obtained from the owners using the Estonian University of Life Sciences’ small animal clinic consent and price calculation form (see Appendix 2; Supplementary material). Participation in the owner survey was voluntary and anonymous, and this was explained in the survey introduction (see Appendix 1; Supplementary material). Owners whose pets were in critical condition or euthanased were not invited to participate in the survey.

## Results

### Owner survey

Altogether, 351 owner questionnaires were filled out: 140 by rabbit owners, 122 by guinea pig owners, and 89 by rat owners. Two hundred and ninety-one questionnaires were completed online and 60 on paper. In addition, a further three paper questionnaires were omitted from the study; two of which had been filled out incompletely while another was completed by the owner of a pet mouse.

**Figure 1.**
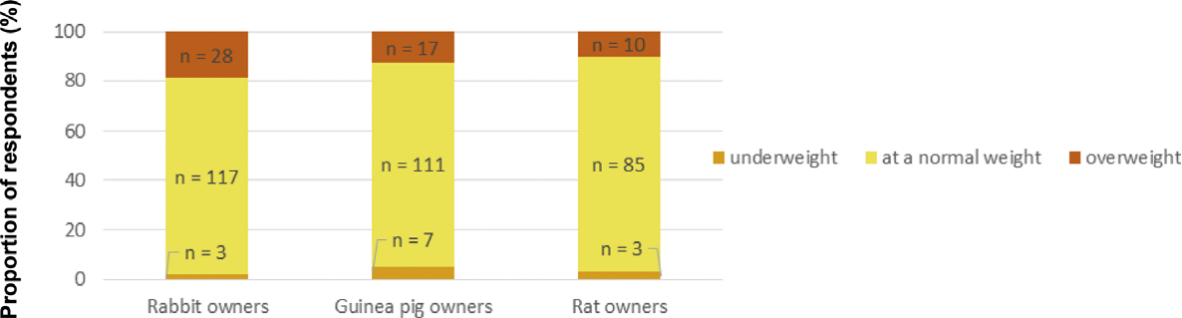
Exotic companion mammal owners’ assessments via questionnaire (n = 351) regarding their pets’ body condition as shown by the number of respondents selecting each option for each surveyed species.

**Figure 2.**
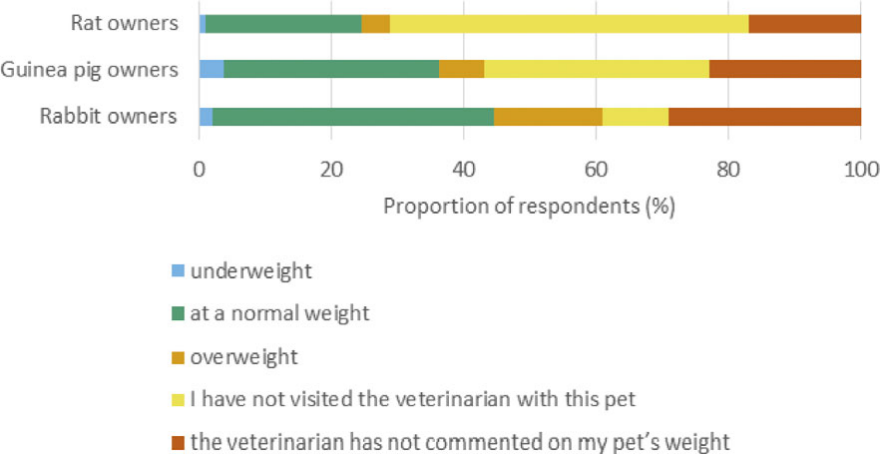
Exotic companion mammal owners’ reported assessments via questionnaire (n = 351) by veterinarians regarding their pets’ body condition as shown by the number of respondents selecting each option for each surveyed species.

**Figure 3.**
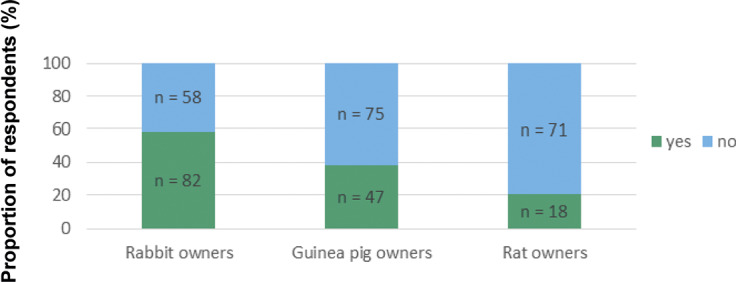
Exotic companion mammal owners (n = 351) who reported via questionnaire having received husbandry advice from a veterinarian as shown by number of respondents selecting each option for each surveyed species.

Of all owners (n = 351), the proportion indicating that at least one of their pets was overweight according to their own judgment was 20% (28) for rabbit owners, 14% (17) for guinea pig owners and 11% (10) for rat owners (Figure 1), whereas 17% (28), 7% (9) and 4% (4) of rabbit, guinea pig and rat owners, respectively, indicated that at least one of their pets had been described as overweight by a veterinarian (Figure 2). Thirty-one percent (43) of rabbit owners, 25% (31) of guinea pig owners and 18% (16) of rat owners had at least one pet whose body condition had not been commented on by a veterinarian (Figure 2). Eleven percent (15), 38% (46) and 57% (51) of rabbit, guinea pig and rat owners, respectively, had at least one pet that had been party to a consultation by a veterinarian. Of all respondents, 58% (204) had not received advice from a veterinarian regarding husbandry or feeding (Figure 3).

**Figure 4.**
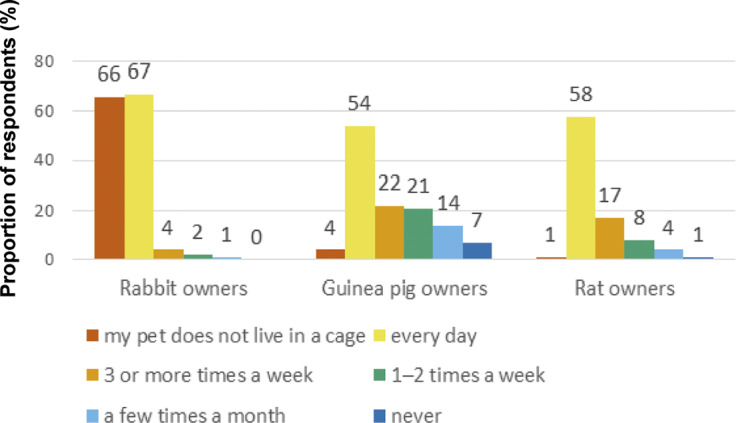
The estimated amount of free-range time outside of the cage that exotic companion mammal owners’ (n = 351) permitted their pets in a questionnaire survey. Number of respondents shown selecting each option for the species in question.

**Figure 5.**
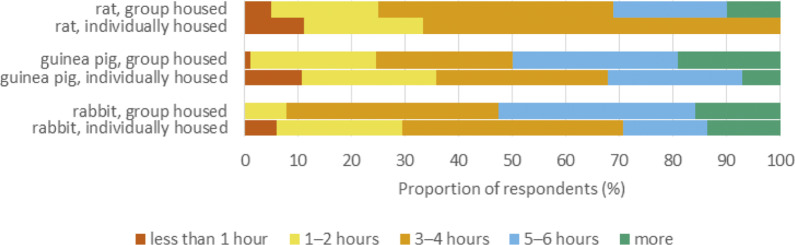
Exotic companion mammal owners’ (n = 351) perceived activity levels of pet species as shown by the proportion of respondents choosing each option, separated by housing type (group- vs individually housed pets).

Twenty-seven percent (38) of rabbit owners, 76% (93) of guinea pig owners, and 89% (79) of rat owners indicated that their pet lived together with at least one other individual of the same species (Figure 4). Forty-eight percent (67), 44% (54) and 65% (58) of rabbit, guinea pig and rat owners, respectively, indicated that they allowed their cage-housed pet free daily roam time outside of their cage, whereas 47% (66), 3% (4) and 1% (1) indicated that their pet was not housed in a cage, i.e. presumably housed free-range (Figure 4). The majority of pet owners described their pets as being active for 3–4 h per day or less (Figure 5) while 14% (20), 16% (20) and 9% (8) of rabbit, guinea pig and rat owners, respectively, stated their pets to be active for more than 6 h per day.

Activity levels did not differ significantly between species (h[2] = 2.82; *P* = 0.245). Each activity level option was given a rank from 1 to 5 and the results were compared for individually and group-housed animals in each species. In all species, animals housed with a conspecific were reported as being more active than those housed individually (Figure 5). This difference was statistically significant in rabbits (z = –2.80; *P* = 0.005) and guinea pigs (z = –1.98; *P* = 0.048), but not rats (z = –1.58; *P* = 0.115).

Different types of feed that owners indicated their pets had *ad libitum* or occasional access to are listed in Tables 1 and 2. Specified under ‘other’, responses included *ad libitum* access to yoghurt, quinoa, and buckwheat (rats) and oatmeal and chewing sticks (rabbits), and occasional access to meat products, chicken, eggs, rice, buckwheat, oatmeal, porridges, veterinary supplemental feeds, ‘various suitable human food items’ as well as ‘everything’ (rats), fresh branches (guinea pigs), and oatmeal (rabbits).

**Table 1.** Proportion (%) of exotic companion mammal owners’ selections of options of feed given *ad libitum* via questionnaire (n = 351), grouped by species

My pet always has access to…	Rabbit owners (n = 140)	Guinea pig owners (n = 122)	Rat owners (n = 89)
hay	100	98	4
hay pellets	11	7	3
dry food (pellets)	31	68	74
dry food (muesli type)	6	19	34
seeds	1	7	31
fresh fruit	4	14	13
dried fruit	1	2	13
fresh vegetables	19	29	18
dried vegetables	1	4	8
dog or cat food	0	0	2
hay-based treats for rabbits or rodents	5	8	11
grain-based treats for rabbits or rodents (e.g. biscuits)	4	3	16
yoghurt drops	1	1	1
fresh grass	11	12	3
mealworms or insects	0	0	15
supplements (vitamins, minerals)	6	13	16
nuts	0	2	13
my pet does not have unlimited access to food	1	2	12
other	1	2	0

**Table 2.** Proportion (%) of exotic companion mammal owners selecting each option of feed given occasionally via questionnaire (n = 351), grouped by species

I occasionally give my pet…	Rabbit owners (n = 140)	Guinea pig owners (n = 122)	Rat owners (n = 89)
hay	19	11	4
hay pellets	14	4	1
dry food (pellets)	46	20	11
dry food (muesli type)	7	6	12
seeds	11	6	45
fresh fruit	61	68	83
dried fruit	24	7	35
fresh vegetables	75	66	75
dried vegetables	14	7	17
dog or cat food	1	0	15
hay-based treats for rabbits or rodents	41	36	21
grain-based treats for rabbits or rodents (e.g. biscuits)	34	21	47
yoghurt drops	5	7	21
fresh grass	54	61	27
mealworms or insects	0	1	46
supplements (vitamins, minerals)	10	27	11
nuts	9	4	61
I do not give them anything extra	0	2	0
other	1	1	10

### Patient survey

Altogether, body condition data were recorded for 177 animals: 71 rabbits, 73 guinea pigs, and 33 rats (Table 3).

**Table 3.** Descriptive statistics of the surveyed exotic companion mammal population by species attained via questionnaire survey (n = 351)

Characteristic	Rabbits (n = 71), number of surveyed patients (proportion of surveyed patients; %)	Guinea pig*s* (n = 73), number of surveyed patients (proportion of surveyed patients; %)	Rats (n = 33), number of surveyed patients (proportion of surveyed patients; %)
females	36 (51)	41 (56)	17 (52)
males	35 (49)	32 (44)	16 (48)
spayed or neutered	27 (38)	9 (12)	1 (3)
intact	44 (62)	64 (88)	32 (97)
mean (± SD) BCS	3.08 (± 0.71)	2.97 (± 0.79)	3.03 (± 0.97)
mean (± SD) weight (kg)	2.03 (± 0.99)	0.93 (± 0.24)	0.42 (± 0.16)
mean (± SD) age (months)	28.9 (± 26.84)	37.21 (± 17.34)	15.84 (± 7.94)
BCS > 3	20 (28)	17 (23)	9 (27)

BCS: body condition score

Twenty-eight percent of rabbits, 23% of guinea pigs and 27% of rats surveyed were given a BCS of 3.5 or higher and were categorised as overweight. Odds ratios for the effects of sex and gonadectomy status were compared (Table 4) with most of the variables failing to have a significant impact on the odds of being overweight for any species (*P* > 0.05). Spay and neuter status was not analysed statistically in rats since only one rat in the sample was neutered. The only significant association was between male sex and excessive bodyweight in rats, although differences in calculation methods meant the confidence interval was not statistically significant (OR = 5.52, 95% CI 0.81–65.84; *P* = 0.046). However, male guinea pigs were found to have increased odds of being underweight (OR = 3.57, 95% CI 1.15–11.88; *P* = 0.012). Mean ages between the BCS 1–3 group and 3.5–5 group were compared using the *t*-test and no statistically significant correlation was seen. Age and BCS were also compared and the only significant association found was in rats with older rats being thinner (*r* [30] = –0.35; *P* = 0.050).

**Table 4.** Comparison of the effects of sex and gonadectomy status on BCS for each exotic companion mammal species as part of questionnaire survey (n = 351)

Variable	Level	BCS 1–3 (n)	BCS 3.5–5 (n)	Odds ratio (95% CI)	P-value
Rabbits					
Sex	male	24	11	1.37 (0.43, 4.46)	0.368, ns
	female	27	9	*	
Age (months)	mean (± SD)	30.37 (± 29.99)	25.15 (± 16.32)		0.465, ns
Gonadectomy status	intact	34	10	*	
	spayed or neutered	17	10	1.98 (0.61, 6.51)	0.152, ns
Guinea pigs					
Sex	male	26	6	*	
	female	30	11	1.58 (0.46, 5.97)	0.300, ns
Age (months)	mean (± SD)	37.52 (± 18.92)	36.18 (± 11.01)		0.782, ns
Gonadectomy status	intact	51	13	*	
	spayed or neutered	5	4	3.08 (0.53, 16.70)	0.121, ns
Rats					
Sex	male	9	7	5.83 (0.81, 65.84)	0.046
	female	15	2	*	
Age (months)	mean (± SD)	15.96 (± 8.89)	15.50 (± 4.41)		0.191, ns

* indicates the non-exposed group (i.e. this refers to the group that is not exposed to the factor that is being analysed, so if the number is for “males” then the non-exposed group would be “females” (not exposed to the risk factor of being male). BCS: body condition score; ns: not significant

## Discussion

This study shows excessive bodyweight to be a prevalent problem among Estonian exotic companion mammals. Obesity in these species may be a result of poor diet combined with suboptimal husbandry and is known to be associated with increased morbidity and mortality. Approximately one-quarter of the ECM species surveyed in this study were overweight. Furthermore, 3% of rabbits, 3% of guinea pigs and 9% of rats could be considered morbidly obese, with a BCS of 5. These results are comparable with previously established tendencies in the UK, where the prevalence of overweight has been 35 and 26% in surveys of pet rabbits (Sweet *et al.* 2013; Thompson *et al.* 2019).

Some studies have found an association between body condition and sex in rabbits whereby female rabbits shower a higher prevalence for being overweight (Courcier *et al.* 2012; Sweet *et al.* 2013). In rats, ovariectomy is associated with a higher body fat percentage and bodyweight compared to unaltered individuals (Ezzat-Zadeh *et al.* 2017). In contrast to those results, sex and neuter status were not found to be associated with excessive body condition, except in rats, where males were more likely to be overweight.

Additionally, it should be noted that approximately one-quarter to one-third of surveyed animals were at a lower than optimal body condition and male guinea pigs had a greater likelihood of being underweight. A UK study also found that anorexia and dental problems, which may be linked, were more prevalent among male guinea pigs (O’Neill *et al.* 2024). Older age was found to have a negative association with body condition in rats, which may be related to an increased rate of neoplasia and other health problems have a disproportionate effect in older rats (Rey *et al.* 2015). In all species, it is likely that the study population was skewed towards individuals that had a health problem, as they would be more likely to be presented to the veterinarian. Here, fifty-seven, 38 and 11% of rat, guinea pig and rabbit owners, respectively, had at least one pet that had never been taken to the veterinarian. In a survey of UK rat owners, 21% reported never having taken their rat to the veterinarian (Neville *et al.* 2021). It is possible that owners of ECMs do not consider it necessary to take apparently healthy pets to the veterinarian for regular check-ups. Other studies have suggested that financial considerations may be the reason owners do not pursue veterinary services, particularly in cases where ECMs were purchased for children (James & Wills 2025).

Most pet owners indicated not having received any feeding or husbandry instructions from a veterinarian with some also stating that their vet had made no comment regarding their pet’s body condition. Those veterinarians having had minimal experience and training with ECMs may fail to recognise excess bodyweight in these pets and may also be hesitant to advise owners regarding feeding and husbandry. A UK survey (Wills & Holt 2020), found the majority of small animal veterinarians to not be confident in their abilities to treat and diagnose small mammals, compared to cats and dogs.

Based on our results, owners are more likely to recognise obesity in their pets than veterinarians, possibly because veterinarians fail to comment or because many pets are not taken to the vet regularly. Sixteen percent of all respondents stated that according to their judgment, at least one of their pets was overweight, whereas only 11% said that at least one of their pets had been assessed as overweight by a veterinarian. Studies in other countries have found that owners are likely to underestimate their pets’ body condition. A survey of rabbit owners in the US found that 92.5% believed their pet to be in optimal body condition and 0.1% believed their pet to be obese, whereas 21.5% reported that their veterinarians had diagnosed their rabbit with obesity (Mayer *et al.* 2017). Another survey in the UK found that 12.1% of owners described their rabbits as overweight (Rooney *et al.* 2014).

In all species, most owners stated that their pets were active 3–4 h per day or less. It is possible that owners may misjudge the time spent active, as ECMs may be active nocturnally or housed outdoors. It is known that in the wild, Norway rats are active for 5–11 h per day (Makowska 2021). Wild rabbits spend the majority of their time above ground engaged in active behaviours, whereas cage-kept rabbits spend the majority of their time inactive (Thurston & Ottesen 2021). The majority of pet owners in all species reported housing their pets in a cage, which may decrease activity levels and place animals at increased risk of gaining excessive weight. It is possible that it may not have occurred to some owners that a hutch is actually a cage. There was also a statistically significant association between being kept with a conspecific and higher activity levels, which is in line with previous research. Individual housing has serious detrimental welfare effects for all species surveyed, but only 27% of rabbit, 76% of guinea pig and 89% of rat owners reported keeping their pet with a companion of the same species. Referring to a variety of UK surveys, 97.6% of rat owners, and 41.9 or 59.3% of rabbit owners reported keeping their pets with a companion; moreover, 78.6% of guinea pigs were reportedly housed with a conspecific (Rooney *et al.* 2014; Harrup & Rooney 2020; McMahon & Wigham 2020; Neville *et al.* 2021).

Rabbits kept in larger pens (3.35 m^2^) have been shown to express a greater variety of behaviours as well as more frequent physical activity compared to those kept in medium (1.68 m^2^) or small (0.88 m^2^) pens, with larger and giant breeds showing a stronger effect of pen size on behaviour (Dixon *et al.* 2010). Enclosure size has also been found to correlate positively with the frequency and diversity of positive behaviours in pet guinea pigs based on owner questionnaires (Harrup & Rooney 2020). Rats housed in multi-level cages that allow climbing and jumping are more confident and easier to handle (Makowska 2021). In laboratory mice, environmental enrichment prevents and reduces binge-like sucrose consumption (Rodríguez-Ortega 2019). Based on this, recommendations for reducing body condition in ECMs may include increasing free-range time outside of the cage (if the animal is kept in a cage) and providing other environmental enrichment.

Isolation has significant detrimental effects on the welfare of guinea pig boars as well as sows, including physiological effects such as higher plasma glucocorticoid levels and decreased complement system activity (Lee 2010). In one particular owner questionnaire study, guinea pigs housed with a conspecific had higher positive behaviour scores compared to guinea pigs housed alone, and those housed with a rabbit had lower positive behaviour scores than those without (Harrup & Rooney 2020). Rats are social animals and tend to benefit from having access to other members of their species (Makowska 2021). Individually housed rabbits display higher levels of fear and a much more narrow range of behaviours than collectively housed rabbits (Trocino *et al.* 2013). Increased oxidative stress, adiposity, hyperinsulinaemia, and higher heart rates have been observed in singly housed rabbits as compared to group-housed controls (Thurston & Ottesen 2021). Veterinarians should advise ECM owners of the welfare and health risks related to individual housing; one manifestation of these may be excessive body condition.

The husbandry questionnaire highlighted feeding practices that do not correspond to the natural diets of ECMs and which may also contribute to excessive weight gain. One hundred percent of rabbit owners and 98% of guinea pig owners stated that their pets always had access to hay. In some cases, respondents ticked an item both in the ‘*ad libitum*’ and the ‘occasional’ category, perhaps reflecting that not all their pets have equal access to the same food items, but most likely representing a misinterpretation of the questions (e.g. in rabbits, where 100% of respondents stated that their pet always has access to hay, but 19% also indicated that their pet is only given hay occasionally). This compares favourably to a survey in the UK, where only 72.8% of guinea pigs received *ad libitum* hay (Wills 2020). However, in another UK-based survey of guinea pig owners, 99.4% fed unlimited hay (Norman & Wills 2016). In one survey, less than 90% of rabbit owners stated that hay makes up the majority of their rabbit’s diet (McMahon & Wigham 2020). Hay should be offered *ad libitum* to both rabbits and guinea pigs, with timothy hay preferred for adult guinea pigs, and alfalfa, which is higher in protein, for young and pregnant or lactating animals (Grant 2014; Clauss & Hatt 2017).

Seventy-five percent of rabbit and 66% of guinea pig owners reported feeding fresh vegetables as a treat; a minority reported unlimited access to vegetables. In previous studies, 77% of British and 98% of Polish guinea pig owners reported feeding vegetables daily. Ninety-nine percent of Polish and 83.2% of UK owners reported feeding fruit (Witkowska *et al.* 2017), whereas in this survey, 61% + 4% of rabbit owners and 68% + 14% of guinea pig owners reported feeding fruit (occasionally + *ad libitum*, respectively). Fruits and particularly grain- or seed-based muesli mixes generally contain too much simple carbohydrates and are too energy-dense for herbivorous ECMs. Guinea pigs are strict herbivores and their wild relatives feed mostly on open grasslands (Quesenberry *et al.* 2012). Sufficient dietary fibre is crucial for guinea pigs in order to maintain dental health, gut motility, caecal pH, and a healthy gut microbiome. Low fibre and excess carbohydrates can cause paralytic ileus, presenting as abnormally soft faecal pellets (Minarikova *et al.* 2015).

Concerningly, a small number of rabbit and guinea pig owners indicated that their pets received unlimited muesli-type feeds. Others also gave grain-based snacks to their pets occasionally. Seed mixes or muesli-type feeds are commonly fed to pet rats, but these may encourage selective feeding and therefore lead to nutritional imbalances or excessive energy consumption. On the other hand, homogeneous pelleted rodent feed may deprive rats of gustatory enrichment and is monotonous in texture (Balcombe 2010). In a 2021 study (Neville *et al.* 2021), over one-third of owners reported feeding their rats home-made diets, which are difficult to assess in terms of nutritional suitability. In our survey, human meals as well as dog or cat food and various treats were commonly fed to rats. In rats and mice, diets high in fat and sugar have been found to negatively affect cognitive functions, such as spatial learning and memory; these deficiencies have been found to appear even earlier than any significant changes in bodyweight (Abbott *et al.* 2019).

The prevalence of overweight in Estonian exotic companion mammals could likely be reduced by educating owners regarding appropriate nutrition and husbandry of these species. The BCS of animals that visit clinics should also be noted down, and explained to the owner, and owners should be given advice regarding the possible causes and welfare consequences of excessive bodyweight. Owners of overweight pets should be instructed to provide their pets with a companion and ample space for exercise; to avoid feeding carbohydrate-rich fruits and treats, especially in rabbits and guinea pigs; and to either reduce the amount of concentrates (pellets, muesli mixes) or simply avoid them altogether, especially in rabbits and guinea pigs.

Further research should be conducted to investigate the status of other exotic pet species kept as companions in Estonia as well as other parts of the world. The conclusions of this study are limited by the small patient and owner sample size. Patients of only one Estonian veterinary clinic were investigated over a period of less than one year. More extensive studies are needed to provide a better representation of pets and owners from all parts of the country. Additional studies should investigate the link between health conditions and body condition as well as whether differences in diet and husbandry are correlated with weight in ECMs. Results may also have been affected by confusion regarding the owner survey. Questions regarding body condition where answers may have differed for individual animals of the same species allowed multiple options to be selected (e.g. a person could tick both ‘overweight’ and ‘underweight’ for the question “I believe that my pet is…”, indicating that they had at least one pet that was overweight and at least one that was underweight), with the intention of including situations where the same owner may keep multiple individuals of the same species under different husbandry or feeding conditions, and to capture all instances where at least one pet in a household had an abnormal body condition. Clinicians often chose to use half-integral scores when rating patients’ body conditions even though scoring guides did not include half points. Since cats and dogs are often scored using a nine-point scale, it may be confusing for non-specialised clinicians to use a different system for exotic pets.

### Animal welfare implications

Results of both the patient and the owner survey point to serious shortcomings in exotic companion mammal welfare in Estonia. Obesity is associated with many negative welfare impacts, but the husbandry and feeding practices that are thought to increase the risk of obesity are also detrimental to animal welfare independently of the excessive bodyweight. Welfare problems are often associated with behavioural signs and the presence of painful clinical diseases; however, a diagnosis of obesity should also be treated as a marker for potential welfare concerns, both those caused directly by obesity as well as those that may correlate with the presence of obesity, i.e. husbandry.

## Supporting information

Pantelejev and Tõnise supplementary material Pantelejev and Tõnise supplementary material

## Supplementary material

The supplementary material for this article can be found at http://doi.org/10.1017/awf.2025.10042.
