# Supplementary material for: Prevalence and awareness of obesity and related husbandry practices in Estonian rabbits, guinea pigs, and rats
Source: Anim Welf. 2025 Oct 10;34:e66. doi: 10.1017/awf.2025.10042 (PMC12554809; doi:10.1017/awf.2025.10042)
Supplement: Pantelejev and Tõnise supplementary material [file S0962728625100420sup001.pdf]

Prevalence and awareness of obesity and related husbandry practices in Estonian rabbits (*Oryctolagus cuniculus*), guinea pigs (*Cavia porcellus*), and rats (*Rattus norvegicus*): Supplementary material

Mariin Pantelejev https://orcid.org/0009-0008-6464-9534, Kristin Tõnise

Estonian University of Life Sciences, FR Kreutzwaldi 1, Tartu 51006, Estonia

Author for correspondence: Mariin Pantelejev, email: mariinpantelejev@gmail.com

Appendix 1. Owner questionnaires (in Estonian and English)

Küsimustik roti-, hiire-, merisea- ja küülikuomanikele

Olen veterinaarmeditsiini tudeng Mariin Pantelejev ja uurin ülekaalulisust Eesti rottide, hiirte, merisigade ja küülikute seas. Järgnev küsimustik on mõeldud loomaomanike teadlikkuse ja hoiakute uurimiseks. Küsimustik on anonüümne ja selle täitmine ei tohiks võtta kauem kui 5 minutit. Kokku on 8 küsimust.

Kui peate kodus mitut nimetatud loomaliikidest, siis valige üks liik, kelle kohta küsimustele vastate. Palun täitke küsimustikku ühe loomaliigi kohta ainult üks kord.

1. Mul on kodus... (kui teil on mitu liiki, valige üks liik, kelle kohta küsimustikku täidate)

○ rott ○ hiir ○ merisiga ○ küülik

2. Arvan, et minu lemmikloom on... (kui teil on mitu samast liigist looma, valige kõik sobivad variandid)

☐ normaalkaalus ☐ alakaaluline ☐ ülekaaluline

3. Olen oma lemmiklooma söötmise ja pidamise kohta saanud juhiseid loomaarstilt.

○ jah ○ ei

4. Loomaarsti hinnangul on minu lemmikloom... (kui teil on mitu samast liigist looma, valige kõik sobivad variandid)

☐ ülekaaluline ☐ alakaaluline ☐ normaalkaaluline

☐ loomaarst ei ole tema kehakaalu kommenteerinud

5. Minu lemmikloomal on kogu aeg kättesaadaval...

☐ hein
☐ heinagraanulid
☐ kuivtoit
☐ seemned
☐ värskeid puuvilju
☐ kuivatatud puuvilju
☐ värskeid köögivilju
☐ kuivatatud köögivilju
☐ koera- või kassitoit
☐ närilistele või küülikutele mõeldud maiustused
☐ jogurtitropsid
☐ värske rohi
☐ ussikesed või putukad
☐ toidulisandid (vitamiinid, mineraalid)
☐ pähklid
☐ minu lemmikloomal ei ole toit kogu aeg kättesaadaval
☐ muud (nimetage) ____

6. Annan oma lemmikloomale aeg-ajalt...

☐ heina
☐ heinagraanuleid
☐ kuivtoitu
☐ seemneid
☐ värskeid puuvilju
☐ kuivatatud puuvilju
☐ värskeid köögivilju
☐ kuivatatud köögivilju
☐ koera- või kassitoitu
☐ närilistele või küülikutele mõeldud maiustusi
☐ jogurtitropse
☐ värsket rohtu
☐ ussikesi või putukaid
☐ toidulisandeid (vitamiinid, mineraalid)
☐ pähkleid
☐ ei anna juurde midagi
☐ muud (nimetage) ____

7. Kas teie lemmikloom elab koos liigikaaslas(t)ega?

○ jah ○ ei

8. Kui tihti lasete oma lemmikloomal liikuda puurist väljas?

○ iga päev ○ mõned korrad nädalas ○ mõned korrad kuus ○ mitte kunagi

Suur tänu vastamast!

Küsimused ja tagasiside on oodatud meiliaadressile mariin.pantelejev@student.emu.ee

Questionnaire for rat, mouse, guinea pig, and rabbit owners

My name is Mariin Pantelejev, I am a student of veterinary medicine and for my final thesis, I am researching obesity among Estonian rats, mice, guinea pigs, and rabbits. The following questionnaire is designed to reflect owners' attitudes and awareness of this topic. The questionnaire is anonymous and completing it should not take more than 5 minutes. There is a total of 9 questions.

If you keep more than one of the abovementioned species, please fill the questionnaire about one species at a time. Please do not submit the questionnaire more than once per species.

1. I have a... (if you have more than one of these species, please choose one of them to answer these questions about)

○ rat ○ mouse ○ guinea pig ○ rabbit

2. I believe that my pet is... (if you have multiple individuals of the same species, please select all that apply)

☐ at a normal weight ☐ underweight ☐ overweight

3. I have received advice regarding the feeding and husbandry of my pet from a veterinarian.

○ yes ○ no

4. According to my veterinarian, my pet is... (if you have multiple individuals of the same species, please select all that apply)

☐ overweight ☐ underweight ☐ at a normal weight

☐ the veterinarian has not commented on my pet's weight

☐ I have not visited the veterinarian with this pet

5. My pet always has access to...

☐ hay

☐ hay pellets

☐ dry food (pellets)

☐ dry food (muesli type)

☐ seeds

☐ fresh fruit

☐ dried fruit

☐ fresh vegetables

☐ dried vegetables

☐ grain-based treats for rabbits or rodents (e.g. biscuits)

☐ hay-based treats for rabbits or rodents

☐ yogurt drops

☐ fresh grass

☐ mealworms or insects

☐ supplements (vitamins, minerals)

☐ nuts

☐ dog or cat food

☐ my pet does not have unlimited access to food

☐ other (please write) ____

6. I occasionally give my pet...

☐ hay

☐ hay pellets

☐ dry food (pellets)

☐ dry food (muesli type)

☐ seeds

☐ fresh fruit

☐ dried fruit

☐ fresh vegetables

☐ dried vegetables

☐ grain-based treats for rabbits or rodents (e.g. biscuits)

☐ hay-based treats for rabbits or rodents

☐ yogurt drops

☐ fresh grass

☐ mealworms or insects

☐ supplements (vitamins, minerals)

☐ nuts

☐ dog or cat food

☐ I do not give them anything extra

☐ other (please write) ____

7. Is your pet housed with other individuals from the same species?

○ yes ○ no

8. How often is your pet allowed free-roam time out of the cage?

○ my pet does not live in a cage

○ every day ○ 3 or more times a week ○ 1–2 times a week

○ a few times a month ○ never

9. How much time would you estimate your pet spends actively moving every day (running around, playing, building a nest etc)?

○ less than 1 hour ○ 1–2 hours ○ 3–4 hours ○ 5–6 hours ○ more

Thank you for your response!

Questions or feedback can be sent to mariin.pantelejev@student.emu.ee.

Appendix 2. General information and consent form for pet owners (in Estonian)

Käesolevaga kinnitan allkirjaga, et:

1. olen looma omanik või otsene valdaja;

1.1.otsese valdaja täiendav kinnitus:

1.1.1. kinnitan, et loom on minu õiguspärases valduses ning looma omanik on mind volitanud otsustama loomaga seostuvate tegevuste üle, sealhulgas volitanud andma käesolevat nõusolekut;

1.1.2. kinnitan, et juhul kui minu ülaltoodud kinnitus ei ole mistahes põhjusel õige, kohustun isiklikult vastutama looma omaniku ees ja vabastama EMÜ

loomakliiniku ning selle töötajad mistahes omaniku poolt esitatavatest nõuetest, sh hüvitama kõik võimalikud kulud seoses oma käesoleva nõusolekuga;

2. olen nõustunud:

2.1. minu/minu poolt esindatava omaniku looma (edaspidi: looma) läbivaatamisega, mida on teinud ja mille tulemustest ja looma terviseseisundist, võimalikest haigustest ja nende kulgemisest on mind mulle arusaadaval moel teavitanud veterinaararst ____ (ees- ja perekonnanimi) (edaspidi: veterinaararst);

2.2. loomale diagnoosimiseks ja raviks soovitatud uuringute ja/või protseduuride (sh anesteesia ja/või kirurgiliste protseduuride) tegemisega, kusjuures uuringute ja/või protseduuride olemusest ja otstarbest, nende osutamisega kaasnevatest ohtudest ja tagajärgedest ning nende hindadest on veterinaararst mind mulle arusaadavalt teavitanud;

2.3. veterinaarmeditsiini eriala üliõpilaste õppe-eesmärgil osalemisega loomale tehtavatel uuringutel ja/või protseduuridel;

2.4. looma terviseandmete kasutamisega teadus- ja uurimustöö eesmärgil;

3. olen veterinaararstile oma parima arusaama järgi avaldanud kõik vajalikud asjaolud seotult looma terviseseisundi ning talle tehtavate uuringute ja/või protseduuridega;

4. olen teadlik veterinaararsti kohustusest informeerida mind uuringute ja/või protseduuride orienteeruva hinna alates 25%-lisest ületamisest;

5. kohustun tellitud uuringute ja protseduuride eest tasuma sularahas või maksekaardiga;

Loomaomaniku / otsese valdaja:

allkirjastamise kellaaeg ____________________________________

allkiri ____

Veterinaararsti allkiri ____

General information and consent form for pet owners (in English)

I, the undersigned, confirm that:

1. I am the owner or direct holder of this animal;

 1.1. additional confirmation for direct holder of the animal:

  1.1.1. I confirm that this animal is legally held by me and that the owner of the animal has authorised me to make decisions pertaining to the animal, including to sign this consent form;

  1.1.2. I confirm that in the event that my abovementioned confirmation is not true for any reason, I am to be personally held responsible to the animal's owner and I will clear the veterinary clinic of the Estonian University of Life Sciences and its employees from any claims made by the owner, including the compensation of any costs related to this consent;

2. I consent to:

 2.1. the clinical examination of the animal owned by me or whose owner I represent (from here onwards referred to as the animal), of whose results, the animal's health status, possible diseases and their course I am to be informed in a manner understandable to me, carried out by the veterinarian, ______________________________ (first and last name) (from here onwards referred to as the veterinarian);

 2.2. the analyses and/or procedures (including anesthetic and/or surgical procedures) which have been recommended for the animal's diagnosis and treatment, of whose nature and purpose, the risks and consequences associated with them, and costs, I have been informed of in a manner understandable to me by the veterinarian;

 2.3. students of veterinary medicine participating, as part of their studies, in analyses and/or procedures carried out with the animal;

 2.4. use of the animal's health information for scientific and research purposes;

3. I have informed the veterinarian to my best understanding of all relevant facts related to the animal's health condition and the analyses and/or procedures to be carried out;

4. I am aware of the veterinarian's duty to inform me in case the predicted cost of the analyses and/or procedures exceeds the estimate by 25% or more;

5. I oblige to pay for the ordered analyses and procedures in cash or by payment card;

The owner's or direct holder's

time of signature ______________________________

signature ______________________________

Veterinarian's signature

____
